# Students’ understanding and support for anti‐racism in universities

**DOI:** 10.1111/bjso.12482

**Published:** 2021-07-13

**Authors:** Glen S. Jankowski

**Affiliations:** ^1^ School of Social Sciences Leeds Beckett University Leeds UK

**Keywords:** racism, students, universities, positive actions, UK

## Abstract

Critical Race Theory (CRT) suggests psychology’s contribution to racism takes various forms. Abstractly, racism is promoted through psychology’s flawed theoretical conceptualization as an individualized, inevitable occurrence. Concretely, it occurs because psychology is one of the most popular reasons students come to university and Black Asian and Minoritized Ethnic (BAME) students report racist harassment and less access to formal support whilst there. This racism and student‐proposed anti‐racist recommendations are often ignored. Concretely assessing what racism students face, assessing how students understand racism, and demonstrating student support for anti‐racist recommendations, are CRT‐informed methods of challenging university racism. White (*n* = 213) and BAME (*n* = 182) UK students were asked about their estimation of racism, any positive action and discrimination experienced, and their access to university support. Participants were also randomized into multiple conditions where five anti‐racist recommendations were proposed (by Professors N. Patel, R. Smith, or no one). Participant consensus was found in high racism estimations, in benefiting from similar positive actions and in accessing four types of university support. However, White students underestimated racism more so, received less discrimination, and reported more access to three university support types. Almost all participants supported the recommendations regardless of proposer. These results suggest the implementation of anti‐racist recommendations converges with university’s interests as student stakeholder support them. Psychologists in universities can advocate for these recommendations and take other anti‐racist actions.

## Background

Recently, the *British Psychological Society* and *American Psychological Association* have acknowledged psychology’s need to confront racism (APA, 2021; BPS, 2020). Confronting psychology’s racism in universities is important for several reasons. First, because psychology is one of the most popular degree choices in the United Kingdom (UCAS, 2019). Second, because psychology contributes to a generalized theoretical minimization of racism (as an individualized prejudicial attitude; Henriques; Leach, 2002; Salter & Haugen, 2017) at least in part through teaching and research that occurs in universities. Finally, students of psychology and other courses, face racist inequalities in universities themselves. Specifically, Black, Asian, Minoritized Ethnic (BAME)^1^ students are 13% less likely to be awarded a high degree classification, are less likely to be employed after graduating, and earn significantly less than their White graduating peers (Khan & Shaheen, 2017; MacGregor‐Smith, 2017; Mance, 2018; NUS & Universities UK, 2019; Pilkington, 2013). For some BAME students, these inequalities are even more pronounced where, for example, Black male graduates face a £4 per hour pay gap (Mance, 2018) and all Black students face a 23% awarding gap (MacGregor‐Smith, 2017; Mance, 2018; NUS & Universities UK, 2019). Some BAME students also report racist harassment on campus, isolation and receiving limited support from staff (Bunce, King, Saran, & Talib, 2019; Eq ualities & Human Rights Commission, 2019; NUS & Universities UK, 2019; Savas, 2014; Stevenson, O’Mahony, Khan, Ghaffar, & Stiell, 2019).

This study follows other research that, broadly, centres racism as an overriding force that shapes human experiences and commits to tackling it. This work includes *Critical Race Psychology* (Salter & Adams, 2013; Salter, Adams, & Perez, 2018; Salter & Haugen, 2017), *QuantCrit* work (Gillborn, Warmington, & Demack, 2018), *Critical Race Metholodogy* (Solórzano & Yosso, 2002) and, most notably, *Critical Race Theory* (CRT; Delgado & Stefancic, 2017). CRT is the collective intellectual anti‐racist roadmap for advocates, including psychologists, that offers various ‘tenets’ to guide anti‐racism. One key tenet of CRT is acceptance that widespread, flawed conceptualizations of racism leave broader racism as ‘*hiding in plain sight*’ (Gillborn, 2019, p. 112). Psychology contributes to this conceptualization in numerous ways. First, social psychological accounts of prejudice position racism as an inevitable occurrence that exists between different groups of people or resides within individuals, failing to account for the power imbalances between groups and the broader nature of racism (Henriques, 1984; Henwood, 1994; Leach, 2002; Salter & Haugen, 2017). Second, psychology presents health, development, socializing and other aspects of human behaviour as uninfluenced by racism despite contrary compelling evidence (Ghavami, Katsiaficas, & Rogers, 2016; McMorris, 1999; Owusu‐Bempah & Howitt, 2000; Paradies, 2006). The BPS’s course accreditation guidance, for example, does not mention ‘race’ or racism at all (BPS 2019). Finally, psychology can explain away racism lending ‘scientific credibility’ (Saini, 2019; Tucker, n.d) to racist, essentialist and deficit narratives that blame BAME people for the inequalities they face (Smith & Hope, 2020; Solórzano & Yosso, 2002). Relatedly, another tenet of CRT is that an individual’s ‘standpoint’ influences how they perceive the world (Delgado & Stefancic, 2017; Park & Liu, 2014). Given western, mainstream, psychology, and higher education is predominately White (whether in psychology samples, psychology editorial boards, BPS presidents or university academics; Arnett, 2008; BMEpsychology.com, 2020; Henrich, Heine, & Norenzayan, 2010; University & College Union, 2013), a flawed conceptualization of racism may produce misunderstandings of racism that proliferate through the discipline unchallenged. In contrast, BAME students and staff may be acutely aware of racism and may possess a unique knowledge of university operations that their White counterparts do not have (such as who can access mitigation and who cannot; Salter & Adams, 2013). This unique knowledge can valuably inform anti‐racism (Aguirre, 2000; Savas, 2014; Solórzano & Yosso, 2002; Yosso, 2013).

Misunderstanding racism can be most prominently seen in a recent nationally representative survey (Mohdin, 2018) that found between 52% and 61% of White British participants reported BAME people faced the same amount or less discrimination in various areas of UK life compared with White people (for similar research in Australia see Leviston, Dandy, & Jetten, 2021). Three examples suggest a flawed understanding of racism specifically circulates in universities (Henriques; Reed, Thompson, Brannick, & Sacco, 1991). First, when researchers (Stevenson et al., 2019) asked universities if they had any anti‐racist positive action policies in place, five responded they had, despite the policies being more likely to benefit White (working class) students. The second example refers to the predominance of racism research that solely assesses the interpersonal experiences of BAME students rather than any material and structural barriers they face (Kanter et al., 2017). The third example refers to universities’ colour‐blind practices of failing to even monitor (let alone redress) BAME students’ access to established support (e.g., mitigation), collectively leaving BAME students (and staff) without redress to the structural disadvantage they contend with (Mahony & Weiner, 2020; Stevenson et al., 2019). A final example comes from research (Cabrera, 2014; Kanter et al., 2017; Spanierman et al., 2008) finding some white university participants downplayed racism as an individualized system that they too, could be victims of, despite nonetheless holding racist views themselves.

Psychology’s flawed conceptualization of racism circulates within universities and CRT reminds us this is a significant barrier to anti‐racism (Delgado & Stefancic, 2017; Gillborn, 2019; P. S. Salter & Adams, 2013; Tate, 1997; Yosso, 2013). CRT urges researchers not only to acknowledge racism, as a structural system embedded across multiple institutions, but also to challenge it (Delgado & Stefancic, 2017; Solórzano & Yosso, 2002; Tate, 1997). To challenge racism, CRT’s offers two key way forward. First, CRT advocates we name and concretely monitor racism so as to eschew colour‐blind resistance (Delgado & Stefancic, 2017; Gillborn, 2019; Tate, 1997; Yosso, 2013). Specifically, CRT advocates encourage BAME people to counter the neoliberal and deficit narratives applied to them for example by naming the discrimination they have experienced (Crenshaw, 2006; Solórzano & Yosso, 2002; Yosso, 2013). Crenshaw also advocates for people to identify the structural advantages that might have benefitted them if ever narrating their success (Kimberlé Crenshaw Showreel, 2012). There is therefore a need to assess White and BAME student’s university support received, and their experience of positive actions and discrimination encountered.

Second, CRT points to the concept of interest convergence to help combat racism. This is the notion anti‐racism occurs only when White powerholders see this as within their own interests (Bell, 1995; Delgado & Stefancic, 2017; López, 2003; Park & Liu, 2014). Whilst university interests are complex and not homogenous across the sector, a case could be made that implementing anti‐racist steps is within university interests. Specifically, if it can be demonstrated that students, as the largest stakeholders within these public organizations, support them (NUS & Universities UK, 2019). Especially, given these recommendations were originally proposed by students (NUS & Universities UK, 2019; NUS, 2012). As such, attending to concrete anti‐racist recommendations directly proposed by students may be a feasible first step in challenging university racism.

The *National Union of Students* (NUS) is arguably the single largest student consultation body that exists in the United Kingdom. Helpfully, it has made the following recommendations to counter university racism: *[Universities should]: Ensure all marking is anonymous (Marking), Ensure curriculums represent people and issues from around the world (Diversification), Offer specific mentoring, workshops & training to BAME students (Mentoring),* and *Offer specific bursaries to BAME students on courses that are under‐represented* (*Bursaries*; NUS, 2012; NUS & Universities UK, 2019). Initial research (NUS, 2015, p. 2) also suggests a final recommendation is also needed: *Offering contextual admissions to BAME students* (*Admissions*).

The above anti‐racism recommendations are based on well‐grounded and comprehensive investigations into racism (McDuff, Tatam, Beacock, & Ross, 2018; NUS & Universities UK, 2019; NUS, 2012) and many recommendations are not new. For example, as evidenced by long standing challenges to curriculum whiteness (Du Bois, 1935; López, 2003; Tate, 1997) and advocacy for ‘race’ based affirmative action (Aguirre, 2000; López, 2003; Savas, 2014). Nonetheless they have so far, largely, been ignored (NUS & Universities UK, 2019; Tackling the ‘BPOC’ Attainment Gap in UK Universities, 2018). This reflects a broader trend to ignore anti‐racist calls. For example, British MP David Lammy (2020) recently counted 201 anti‐racist recommendations made since 2017 that the UK Government has ignored (despite originally commissioning the investigations that produced the recommendations itself). The recommendations arose from investigations into racism in various British institutions including the workplace, the criminal justice‐ and immigration‐systems. Understanding resistance to such calls is necessary. Gardner and Ryan (2020) offer evidence that the standpoint of the proposer is again relevant to anti‐racism. The researchers found that when participants (75% White) read a workplace diversity proposal they were more likely to support it if their hypothetical co‐worker was White compared with Black.

### The present study

Psychology’s flawed conceptualizations of racism circulates within universities and, according to CRT (Delgado & Stefancic, 2017), impedes anti‐racism. Anti‐racism in universities is more likely to be implemented if support from students is demonstrated according to CRT’s interest convergence principle (Park & Liu, 2014). Addressing how university students understand racism, eschewing colour‐blindness by assessing student’s positive actions and discrimination encountered and assessing student support for anti‐racism (whilst contextualising this by assessing standpoints) are CRT informed steps that ‘make good’ on psychology’s anti‐racist commitments. This study therefore aims to answer the following questions: (1) How do UK students understand racism? (2) What positive actions, discrimination and university support have UK students experienced? and (3) Do students support university anti‐racist recommendations and does this differ according to the proposer’s ‘race’?

## Method

### Design

This mixed‐methods, cross‐sectional, survey follows the ‘QuantCrit’ approach to research (Garcia, López, & Vélez, 2018; Gillborn et al., 2018) which acknowledges, in particular, the limitations of quantitative methods in capturing the impact of intersectional racism (Gillborn et al., 2018) but argues if research foregrounds BAME people’s experiences, focuses on social justice, critically interprets data as one proxy of experience among others and adds ‘voice’ by including qualitative data it can be valuable.

### Procedure

*Prolific* (the research management company) was utilized in June 2020, to invite eligible participants (current UK university students) to complete the survey. *Prolific* is a company that connects potential participants to researchers and focuses on paying participants a minimum wage in contrast to other recruitment platforms (Peer et al., 2017). Like many forms of practical recruitment strategies (including in‐person recruitment), recruiting from this platform will have disadvantages and advantages. Disadvantages might include systematic and slight differences in responses between participants across various online platforms including *Prolific* (on the topic of food knowledge; Armstrong et al., 2021) and higher naivety specifically for *Prolific* participants (Peer et al., 2017). Potential advantages might include producing more honest response from more diverse participants compared with other platforms (Peer et al., 2017). Steps were taken to mitigate recruitment bias, including having clear, pre‐screening criteria, emphasizing incentives are allocated regardless of response and including a clear attention check question to filter out spam or ineligible participation.

A pre‐determined number of study spaces (*N* = 400) were purchased and participants who indicated they were currently in the United Kingdom and were current students were invited (*N* = 7,531) to participate on a first come first served basis. One survey was sent to participants indicating their ethnicity was White and another to those indicating their ethnicity was Black, Asian, or other minoritized ethnicity [those indicating their ethnicity was ‘other’ (but unspecified) were not invited]. Fifty‐four (19 BAME and 35 White) submissions timed out where participants took a place but did not initiate the survey. These places were reallocated to other participants. Recruitment completed within 10 hours. Within the survey, participants were randomized to 1 of 3 anti‐racist recommendations conditions. The conditions were identical except those in the two experimental conditions were presented a ‘recommendation vignette’ by a Professor Nadiya Patel or Professor Rebecca Smith in the following format: *A researcher, Professor Nadiya Patel, has studied university processes and made a list of recommendations to reduce racism. Please indicate the recommendations you think should take place*. Those in the control condition were presented with the recommendations anonymously (i.e., “A list of recommendations to reduce racism have been made…”). After the survey, participants were debriefed and compensated with credits.

### Participants

There were 395 participant responses (age *M* = 23.21 *SD* = 5.37, range = 18–49), with around half identifying as White (*n* = 213, 54%) and the remainder as BAME (*n* = 182, 46%). Specifically, BAME participants identified as Asian (*n* = 113), Black (*n* = 32), Mixed (*n* = 15), Hispanic (*n* = 11), Arabic (*n* = 7), or ‘Other’ (*n* = 4). Most indicated they were women (*n* = 261, 66%), British (*n* = 320, 81%), heterosexual (318, *n* = 80%), and not disabled (93%). Participants came from a range of UK universities (*n* = 110; the UK currently has 165 universities; Universities UK, n.d.) and had studied, on average, slightly over 2 years.

### Measures

#### Definition of racism

Participants were asked to select which of the following two definitions, provided by DiAngelo (Dastagir, 2018, paras. 11–13) were more accurate: (1) “*Racism is an individual who consciously does not like people based on race, and intentionally seeks to hurt them*” (Individual Racism Definition) and (2) “*Racism is a group’s collective bias backed by legal authority and institutional control*” (Institutional Racism Definition). Participants were able to input their own free‐text response as well by selecting ‘Other’.

#### Racism estimation

Eight questions taken from Mohdin's (2018) nationally representative racism survey were used to assess participant’s estimation of racism. Participants were asked to indicate the level of discrimination for BAME people regarding (1) *Access to University,* (2) *Access to Good Schools,* (3) *In TV Shows or Films?* (4) *Access to Finance?* (5) *In the Workplace?* (6) *In the News?* (7) *Access to Jobs?* Participants were asked to answer on a 3‐point Likert response ranging from −1 (*Less*) to 0 (*No difference*) to +1 (*Greater*). Participants were also asked to respond on a 3‐point Likert scale ranging from 0 (*None at all*), to 3 (*A great deal*) for the eighth item: (8) *To what extent they thought racism was present in UK society today?* After excluding unsure responses, scores across the 8 items were averaged with higher, positive, scores indicating greater perceived racism.

#### Racism accuracy

Nine items were constructed to assess participants’ specific estimation of racism in higher education and wider society. Each item was constructed based on a publicly available source demonstrating relatively recent, numerical, inequalities between White British and BAME British people (full sources are provided in Table 3). Participants were asked to estimate: (1) *What percentage of hate crimes were recorded as race‐related in the United Kingdom in 2017?* (2) *How much more likely (out of 10) a Black person had of being stopped and searched relative to a White person?* (3) *What percentage of BAME people will be invited to a job interview relative to a White person?* (4) *How much more likely, were innocent Black people wrongfully convicted compared to innocent White people on a 10‐point scale from 0 (equal likelihood), to 10 (ten or more times more likely)* and finally the percentage of (5) *White,* (6a) *BAME* and (7b) *Black students who were awarded a 1st or 2:1 grade in Higher Education, respectively*. Scores on item 5 (White attainment) were subtracted from items 6 (BAME attainment) and 7 (Black attainment) to calculate participants’ estimation of a BAME‐ (6b) and Black‐ attainment gap (7b), respectively. Participants’ responses were subtracted from the actual inequality data to form Racism Accuracy scores for items (1–4 and items 6b and 7b). Finally, these item accuracy scores were converted to percentages and then averaged. Scores ranged from −100 to +100. Negative scores indicated an under‐estimation of racism, scores close to 0 indicated accurate estimations, and positive scores indicated an over‐estimation of racism.

#### Positive action and discrimination

Participants were asked about any positive action and discrimination encountered during their education and employment. For positive actions, participants were presented with the following: *Kimberlé Crenshaw argues people should identify the positive and negative discrimination they may have faced in their lives. The author (GJ, 30; a gay, white man who grew up in foster care) can think of 4 examples of positive action he has received relevant to his education experience (and later career):* (1) *a contextual admission (where the university lowered the entry tariff),* (2) *state financial support to continue in education at A Levels (called the Education Maintenance Allowance),* (3) *state support towards living costs for university, and* (5) *state support to buy furniture and equipment when living independently*. For discrimination, participants were presented with a similar version of the above except about the author’s own experience of discrimination (under the homophobic school legislation: Section 28). For both questions, participants were asked if they had experienced positive actions and discrimination (Yes, No, and Unsure), and if they were comfortable sharing this, what specifically these were (using open‐ended responses).

#### University support

Participants were asked to indicate their agreement with the following seven statements designed to assess students’ access to key areas of university support: (1) *I have been or will be on a placement that helps my degree/career (Placement),* (2) *I feel able to gain mitigation, suspension or other form of support for assessments during university (Assessment Support),* (3) *I feel I can access university mental health support (Mental Support),* (4) *I feel I can access university financial support (Financial Support),* (5) *I feel able to share my perspective with lecturers and staff (Share Perspective),* (6) *I have been or will be given positive references from university staff for future employers (Employment Reference),* and (7) *I feel university staff treat me equally to other students (Equal Treatment)*. Participants were asked to respond on a 5‐point scale ranging from −2 (Strongly disagree) to 0 (Neither) to +2 (Strongly agree). Responses were averaged across the statements with higher and positive scores indicating more university support and lower, negative, scores indicating less support.

#### Open‐ended responses

Participants were asked if there was anything else, they would like to add, and if they could not think of anything, to ‘*Please write down a question they thought should have been included in the survey*’.

#### Anti‐racist university recommendation support, ranking, and favourability

Participants read 1 of the above ‘recommendation vignettes’ and were then presented with the five anti‐racist recommendations: *Marking, Diversification, Mentoring, Admissions,* and *Bursaries*. Participants were then asked to rank the recommendations that should take place in priority order of importance from 1 (*Most important*) to 6 (*Less Important*), leaving blank any recommendations that should not take place. Participants were able to input their own free‐text response as well by selecting ‘Other’. Responses were collapsed into three categories (1) *More important* (1st, 2nd, and 3rd ranking), (2) *Less important* (4th, 5th, and 6th ranking), and (3) *Not selected* (Blank responses). Participants were also asked to rank collectively how (1) *Useful*, (2) *Appropriate,* and (3) *Important* these recommendations were from 0 (*lowest*) to 10 (*highest*). Scores across these latter three questions were averaged with higher scores indicating greater recommendation favourability.

#### Anti‐racism university combatting

Participants were asked to what extent they thought universities should combat racism by selecting one of the following responses: (0) *Universities should not combat racism (e.g., because it is not their job)*, (1) *Universities should combat racism that occurs in Higher Education (HE)*, (2) *Universities should combat racism that occurs in HE & Employment,* and (3) *Universities should combat racism that occurs in HE & Employment & Society*. Scores were averaged with higher scores indicating that universities should combat racism to a greater extent.

## Results

### Data screening and baseline equivalence of conditions

Missing data were minimal (the highest number of missing values was *n* = 12 (3%) on the penultimate ‘Equal treatment’ question) and appeared to be random (the Little MCAR test was non‐significant; *χ* ^2^ = 673.18 (625), *p* = .088). Five participants were excluded given they appeared to be significant multivariate outliers (and nonsensical responses; *p*’s < .05). Finally, White participants did not differ to BAME participants in their study duration, demographics or distribution across conditions (*p*’s > .08).

### Analytic strategy

Descriptives, frequencies, and comparative statistics from other sources are presented in Tables 1, 2, 3, 4. Inferential statistics are presented in the following text from three between‐subjects MANOVAs and two chi‐squares where space permits. For these analyses, the more robust Pillai’s test and Bonferroni corrected *p*‐values were interpreted (Field, 2009). Confidence intervals of the mean differences between the groups are also reported (equal variances unassumed). Partial eta‐squared and Cramer’s V statistics served as effect sizes.

**Table 1:** Frequency (%) of participants’ estimation of discrimination towards BAME people in seven different areas of UK life and in general from the present study (*N* = 391) compared with responses by participants from Mohdin (2018)^a^

Item name	BAME discrimination extent in seven different areas of UK life							
	Present study participants				Mohdin (2018) participants			
	Lesser	No difference	Greater	Unsure	Lesser	No difference	Greater	Unsure
University access	10	25	57	8	15	47	20	18
Good school access	10	18	67	5	14	46	23	17
In TV shows or films	10	18	66	6	20	40	23	17
Financial access	8	22	54	16	10	47	17	26
In the workplace	10	14	71	6	18	36	29	17
In the news	7	14	71	8	16	36	30	18
Access to jobs	9	14	69	8	17	3	31	17
Average	9	18	65	8	16	36	25	19

	Racism extent generally									
	Present study participants					Mohdin (2018) participants				
	None at all	A little	Somewhat	A great deal	Unsure	None at all	A little	Somewhat	A great deal	Unsure
Extent of UK racism	1	10	35	55	0	1	20	52	20	6

Note

^a^ Mohdin, A. (2018, December 20). Up to 40% of Britons think BAME people do not face more discrimination. The Guardian.http://www.theguardian.com/world/2018/dec/20/up‐to‐40‐of‐britons‐think‐bame‐people‐do‐not‐face‐more‐discrimination

**Table 2:** Descriptives indicating participants’ estimation and accuracy responses regarding racism

	White	BAME	Total	Correct response	Accuracy (Total response –Correct response)
	*M* (*SD*)	*M* (*SD*)	*M* (*SD*)		*M* (*SD*)
2017 Race Hate Crimes (%)	52 (22)	60 (22)	55 (22)	76^a^	−21 (22)
2016/17 Stop & Search (/10)	6 (2)	7 (2)	6 (2)	8^b^	−2 (2)
2016 Conviction rate (/10)	5 (2)	6 (3)	6 (2)	7^c^	−1 (2)
Job Interview (%)	35 (23)	36 (21)	36 (22)	50^d^	−14 (22)
2018 BAME Attainment Gap (%)	18 (18)	17 (20)	18 (19)	13^e^	5 (19)
2018 Black Attainment Gap (%)	25 (21)	27 (22)	26 (21)	23^e^	3 (21)
Average Racism Accuracy score^f^	−9 (9)	−5 (10)	−7 (10)		

Note

^a^ Source: Home Office. (2017). Police powers and procedures, England and Wales, year ending 31 March 2017 (p. 52).https://assets.publishing.service.gov.uk/government/uploads/system/uploads/attachment_data/file/658099/police‐powers‐procedures‐mar17‐hosb2017.pdf

^b^ Source: Home Office. (2018). Hate Crime, England, and Wales, 2017/18 (p. 40).https://assets.publishing.service.gov.uk/government/uploads/system/uploads/attachment_data/file/748598/hate‐crime‐1718‐hosb2018.pdf

^c^ Source: National Registry of Exonerations. (2017). *Race and Wrongful Convictions in the United States* (p. 37).http://www.law.umich.edu/special/exoneration/Documents/Race_and_Wrongful_Convictions.pdf

^d^ Source: Zschirnt, E., & Ruedin, D. (2016). Ethnic discrimination in hiring decisions: A meta‐analysis of correspondence tests 1990–2015. Journal of Ethnic and Migration Studies, 42(7), 1115–1134.https://doi.org/10.1080/1369183X.2015.1133279

^e^ Source: NUS, & Universities UK. (2019). Black, Asian, and Minority Ethnic student attainment at UK universities: #closingthegap (p. 88). Universities UK.https://www.universitiesuk.ac.uk/policy‐and‐analysis/reports/Pages/bame‐student‐attainment‐uk‐universities‐closing‐the‐gap.aspx?fbclid=IwAR1SyhMVElMJURVupBDArtLblh4kyWLCzi8X‐p7HBIthFD722obG93bQKds

^f^ Calculated as average of the following: BAME Attainment Accuracy, Black Attainment Accuracy, Stop & Search Accuracy (after percentage conversion), Job Accuracy, Conviction Accuracy (after percentage conversion) and Race Hate Crime Accuracy.

**Table 3:** Descriptives indicating levels of agreement in accessing university support

	White	BAME	Total
University Support Type	*M* (*SD*)	*M* (*SD*)	*M* (*SD*)
Placement	0.58 (1.37)	0.44 (1.24)	0.51 (1.31)
**Assessment Support**	**0.78 (0.86)**	**0.51 (1.04)**	0.65 (0.96)
Mental Support	0.94 (0.97)	0.72 (1.12)	0.83 (1.05)
Financial Support	0.51 (1.08)	0.37 (1.16)	0.45 (1.12)
**Share Perspective** Employment References	**0.70 (1.03)** 0.93 (0.92)	**0.35 (1.16)** 0.85 (0.85)	0.54 (1.10) 0.89 (0.89)
**Equal Treatment**	**1.14 (0.85)**	**0.86 (0.93)**	1.02 (0.90)

Note

Response range −2 (Strongly Disagree) to 2 (Strongly Agree).

Bold indicates significant between group difference with a Bonferroni corrected *p* value of *p* =.007.

**Table 4:** Percentage agreement and importance scores of participants’ Anti‐Racist Higher Education Recommendations responses

	Percentages of participants that believe recommendations should take place (%)	Importance ranking (lower scores indicate greater importance)
		*M* (*SD*)
Diversification	97	2.09 (1.18)
Marking	98	2.30 (1.49)
Bursaries	90	3.15 (1.27)
Mentoring	92	3.46 (1.28)
Admissions	87	3.62 (1.28)
Other	‐	4.85 (1.70)

### Racism extent and racism accuracy

More participants selected the *Individual* definition of racism (50%) compared with the *Institutional* (42%). The remaining 8% selected *Other* which tended to be a combination of both definitions. Most participants estimated BAME discrimination in the seven different areas of UK life as *Greater* (54%–71%) followed by *No difference* (14%–25%) and *Lesser* (7%–10%). Participants differed to Mohdin’s (2018) by rating *Greater* discrimination more frequently. However, participants in both studies were similar in the pattern of their estimations across the 7 UK areas of life. Most participants estimated the extent of racism present in UK society generally as *A great deal* (*n* = 213, 54.5%) which was higher than Mohdin’s (2018; 20%). See Table 1 for full frequencies across both studies. On average, participants underestimated the extent of racism, across the *Racism Accuracy* items (e.g., *Black attainment*), by −7% (see Table 2).

For the second MANO*V*A, there was a significant between‐subjects multivariate main effect across the two groups (*V* = 0.03, *F*(2,388) = 2.00, *p* = .003, *partial n^2^* = 0.03). Univariate analyses revealed that *UK Racism Extent* did not differ between the two groups (White: *M* = 0.57, *SD* = 63; BAME: *M* = 0.63, *SD* = 0.54, *F*(1, 389) = 0.97, *p* =.319, *partial n^2^* = 0.003, CI [−0.16, 0.05]). However, *Racism Accuracy* did, where White students underestimated racism to a greater extent (*M* = −8.63, *SD* = 9.23, *n* = 210) compared with BAME students (*M* = −5.23, *SD* = 10.35, *n* = 181; *F*(1, 389) = 11.80, *p* = .001, *partial n^2^* = 0.029, CI Diffs [−5.37, −1.44]).

### Positive action and discrimination

A quarter of participants (24%) indicated they had experienced positive action in relation to their education and employment. Forty‐eight participants detailed what this positive action was. These were gaining: financial support for education (*n* = 22), a contextual admission (*n* = 8), mentoring to attend university (*n* = 7), a job or promotion (*n* = 5), disability adjustments or equipment (*n* = 2), having positive interpersonal interactions (*n* = 2), mitigation (*n* = 1), and a conference travel grant (*n* = 1). When it was identified (*n* = 44), this positive action was described as likely arising on the basis of participant’s class status (e.g., low income, state school attendance *n* = 26), ‘race’ (*n* = 5), gender (*n* = 4), class and ‘race’ combined (*n* = 4), disability (*n* = 3), and sexuality (*n* = 2).

A third (35%) of participants indicated they experienced discrimination. Of the 41 who detailed the experience, this was commonly bullying/isolation (*n* = 16), teacher discrimination (including writing students off, not offering assignment support or being ignorant about religious practises; *n* = 8), interpersonal sexism (such as being interrupted, unwanted contact and overhearing sexist stereotypes; *n* = 6), non‐specific discrimination (*n* = 4), job hire or promotion discrimination (*n* = 4), having to study under homophobic public body legislation (*n* = 2) and being stalked when shopping (*n* = 1). When it was identified (*n* = 40), this discrimination was described as likely arising on the basis of participant’s ‘race’ (*n* = 16), gender (*n* = 8), nationality (*n* = 4), Islamic faith (*n* = 4), sexuality (*n* = 3), religion (*n* = 1), class and ‘race’ combined (*n* = 1), ‘Whiteness’ (*n* = 1), class (*n* = 1), and disability (*n* = 1).

A 2 × 3 × 2 chi‐square analysis was also conducted to determine whether participants (White, BAME) differed in their responses (Yes, No, Unsure) in number of *Positive Action* or *Discrimination* experiences. This revealed no significant differences between the groups on the number of positive actions experienced [White: *n* = 47 (22%) vs. BAME: *n* = 45 (25%); *χ* ^2^ (2) = 2.30, *p* = .316, *Cramer’s V* = 0.08]. There were however significant differences between the groups on the number of discriminations experienced [*χ* ^2^ (2) = 30.47, *p* < .001, *Cramer’s V* = 0.28]. The standardised residuals revealed fewer White participants (*n* = 48, 23%, *z* = −3.0) reported discrimination compared with BAME participants (*n* = 88, 50%, *z* = 3.3).

### Access to university support

Between one‐half and three‐quarters of participants indicated agreement (either Strongly or Somewhat) in accessing the following types of university support: *Equal Treatment* (77%), *Mental Support* (74%), *Employment Reference* (72%), *Assessment Support* (64%), *Share Perspective* (63%), *Placement* (58%), and *Financial Support* (55%).

For the final MANO*V*A, there was a significant between‐subjects multivariate main effect across the two groups (*V* = 0.05, *F*(7,371) = 2.64, *p* = .011, *partial n^2^* = 0.05). Univariate analyses revealed that White students rated higher agreement that they could access *Assessment Support* relative to BAME students (*F*(1,377) = 7.69, *p* = .006, *partial n^2^* = 0.020, CI [0.47, 0.79]), could *Share Perspectives* compared to BAME students (*F*(1,377) = 9.30, *p* = .002, *partial n^2^* = 0.024, CI [0.11, 0.56]) and received *Equal Treatment* compared with BAME students (*F*(1,377) = 9.67, *p* =.002, *partial n^2^* = 0.025, CI [0.10, 0.47]). There were no differences in access to *Placements, Mental Support, Financial Support*, or *Employment References* (*p*’s > .40). See Table 3 for complete descriptives.

### Open‐ended responses

A total of 202 participants (52%) responded to this question with some leaving multiple responses (*n* = 229). In line with the study aims, these responses were approximately categorized into three patterns below [except 12 (5%) which were about survey edits such as spelling corrections] indicating support or issues with anti‐racism (either as it was conceived in the study or more broadly). Further subcategories were also produced. Example responses to these open‐ended questions are available in Table 5.

1. Responses indicating explicit support for anti‐racism (*n* = 50)

**Table 5:** Example participant responses to the open‐ended question categorized and subcategorized: ‘Was there anything else/ a question you think should have been included in this study?’

Response (sub)categorized as…		
…explicit support for anti‐racism (*n* = 50).	…implicit support for anti‐racism (*n* = 153).	…explicit or implicit issues with anti‐racism (*n* = 14).
*(1a) ‘Thank you for the study. I hope studies like these will bring about positive change’*.	*(2a) ‘Possible question: level of diversity in the area where the participant grew up’*.	*(3a) ‘Yes, I detest the separation of people by their skin colour. Asking anecdotal questions will surely skew the results’*.
*(1b) ‘More specific ways on how racism can be tackled in HE’*.	*(2e) whether students have experienced discrimination from other students*	*(3a) ‘I can’t help feel a palpable politic bias from these questions. I worry that university students can’t escape identity politics, and surveys like this one are an example of this’*.
*(1c) ‘I think more research like this should be done. My course…have just sent an email saying what they plan to do but ideally it should be backed by research’*.	*(2h) ‘Do you think you have ever been racist towards anyone? Would you be able to describe this situation?’*	*(3b) ‘As a BAME individual, I will always be grateful that my parents chose this country to move to, rather than any other. I strongly feel almost every society globally has an inherent disliking for any individual whose appearance is diferent to the natives, but that this prejudice is very small in this country compared to others’*.
*(1c) ‘Sometimes I'm unsure whether all the lowered admission requirements or extra funding/quota for minorities is all that good of an idea. I'm generally supportive of those policies, of course, but far too often I've seen them used by people from the right as ammunition of how unfair it is, how it's now the white people who are discriminated etc. I'm minority as well’*.	*(2m)'What percentage of black women have less job opportunities compared to black men?’*	*(3c)'My uni creates a good environment for all races’*.

(1a) Twenty‐eight participants indicated they supported anti‐racism (e.g., by noting ‘Black Lives Matter’), were keen to learn more about anti‐racism and were looking forward to receiving the survey results. (1b) Twelve suggested anti‐racist recommendations they wished would take place (such as curriculum diversification, university outreach at younger ages, more BAME leaders). (1c) Six indicated general support for positive actions but concern it could be misinterpreted as ‘reverse racism’, could lead to perceptions of tokenism or may arise without proper research backing it up. (1d) Finally, some participants indicated the problematic homogenization of people into the category BAME noting it obscured the important differences and forms of racism that affected those within this group (*n* = 4).

- Responses indicating implicit support for anti‐racism (n = 153)

Questions for further research were suggested about (2a) how racially diverse participants experiences were (*n* = 19), (2b) racism from staff and universities (*n* = 18), (2c) witnessing racism (*n* = 18), (2d) the causes, extent, and prevalence of racism (*n* = 14), (2e) personally experiencing racism (*n* = 11), (2f) racism from fellow students (*n* = 11), (2g) anti‐racist recommendations (*n* = 9), (2h) personally being racist themselves (*n* = 6), (2i) racism in employment and housing (*n* = 6), (2j) unconscious or covert forms of racism (*n* = 4), and (2k) colourism (*n* = 2). Participants also suggested questions about (2l) racism’s intersection with class (*n* = 14), and (2m) gender (*n* = 3) and questions about other discriminations involved with 2l) class (*n* = 4), (2n) being LGBT (*n* = 4), (2o) disability (*n* = 2), and (2p) immigration (*n* = 1).

- Responses indicating explicit or implicit issues with anti‐racism (n = 14)

(3a) This included participants who perceived political bias where the research was believed to highlight racism where it did not exist (*n* = 2). (3b) Eight participants also indicated that they believed the United Kingdom to largely be a meritocracy, to harbour no/ minimal racism, and racism to be an inherent feature of human nature in any case. (3c) Finally, four BAME participants indicated they experienced no or minimal racism in the United Kingdom or their university.

### Anti‐Racist higher education recommendations and combatting

Most participants indicated they believed the recommendations should take place (87%–98%) and rated them collectively as highly favourable (*M* = 7.57, *SD* = 1.94). Just 16 (4.3%) participants rated the recommendation’s favourability as low (≤3). In order of most important, participants ranked the recommendations as follows: *Diversification, Marking, Bursaries, Mentoring, Admissions,* and *Other*. Full descriptives are provided in Table 4. Most participants indicated universities should combat racism broadly in *Higher Education, Employment, and Society* (81%). Twenty participants made other anti‐racist recommendations including having robust racist harassment policies (*n* = 6), removal of any racist students or staff (*n* = 3), bursaries based on low income/ and ‘race’ for students (*n* = 3), more BAME staff (*n* = 3), mentoring (*n* = 2), anonymising ‘race’ in admissions (*n* = 1), offering anti‐racist mental‐health support (*n* = 1), and education about anti‐racism (*n* = 1). Finally, five participants indicated universities should not take anti‐racist actions and should instead ‘treat everyone equally’ (*n* = 5).

A 6 × 3 × 5 chi square assessed whether participants (White‐Nadiya, White‐Rebecca, White‐Control, BAME‐Nadiya, BAME‐Rebecca, BAME‐Control) differed in their importance ranking (*Higher, Lower,* and *Not*) on any of the recommendations (*Marking, Diversification, Mentoring, Admissions,* and *Bursaries*). No difference between the groups, in any condition, on any of the recommendations were found (*p*’s >.05). Finally, there was not a significant between‐subjects multivariate main effect across the two groups meaning neither White nor BAME participants differed in their Favourability or Combatting responses in any condition (*p*’s >.05).

## Discussion

This study sought to assess how university students understood racism, what support and positive action they access and whether they support anti‐racist recommendations in response to recent anti‐racist commitments from mainstream psychology organizational bodies (APA, 2021; BPS, 2020) and informed by CRT (Delgado & Stefancic, 2017; Salter & Adams, 2013).

### Racist estimation and accuracy

Half the participants selected the individual definition of racism. Participants were more accepting of racism’s occurrence compared with those in the UK survey (Mohdin, 2018). Furthermore, participants were very accurate in their estimation of the BAME‐ and Black‐ attainment gaps. Nonetheless, there was a slight under‐estimation across the racism items by around −7% and for some items (e.g., racist hate crimes) this increased to an under‐estimation of −20%. White students slightly under‐estimated racism more so than BAME. This supports previous research findings (Howarth, 2009) and CRT (Delgado & Stefancic, 2017) in highlighting that those who have lived experience of racism are more knowledgeable about it. Nonetheless, the difference was slight, and participants estimated more racism than other samples (Mohdin, 2018). These findings can be taken in two ways, first that many students (especially BAME students) appear to be aware of racism, especially in its varied forms. It may be that Black Lives Matter protests in the Summer of 2020 (Dave et al., 2020) increased student anti‐racist understanding, and further, that this knowledge is underestimated by universities. Alternatively, despite an awareness of racism, the individualization—and an underestimation—of it is still a problem. CRT proponents (Crenshaw, 2006; Delgado & Stefancic, 2017) and others (Dastagir, 2018; Reed, 2008; Salter & Adams, 2013) have criticized psychology’s promotion of an individualized definition for implying that any group can experience racism and implying that individual solutions will suffice. Furthermore, on average 9% of participants reported they were unsure about levels of discrimination in some areas of UK life and reported the least racist discrimination occurred through financial access (as did participants in Mohdin, 2018). In addition, a small minority of the open‐ended responses (6%) did not indicate support for anti‐racism; sometimes reaffirming colour‐blindness or the meritocracy of society. Some of these responses echoed the ‘majoritarian minority story’ (Solórzano & Yosso, 2002), where BAME people feel particularly compelled to defend the status quo and emphasize society is a meritocracy. These findings perhaps speaks to the failure to see racism as a materially unjust system, one that overlaps heavily with class, where BAME people are more likely to earn less, have less inherited wealth and less ‘liquid’ wealth (Khan & Shaheen, 2017; Ladson‐Billings & Tate, 1995; Tippett, Jones‐DeWeever, Rockeymoore, Hamilton, & Darity, 2014).

Pragmatically, the need to counter colour‐blindness and other conceptualizations that leave racism as a minimal, singular system is underscored (Delgado & Stefancic, 2017; Gillborn & Ladson‐Billings, 2010). As Salter and Haugen (2017, p. 124) note: ‘*the psychology of racism is best understood as the reproduction, maintenance, and internalization of preexisting, historically derived, systemic racial dynamics regardless of individual‐level racial animus*’. This supports one recommendation in particular: decolonization of the curriculum (Du Bois, 1935; NUS & Universities UK, 2019). Psychology educators can contribute significantly to this. Even in the current accrediting guidance, there is space for curricula to include content on structural racism, e.g., through the BPS’s subtopics that relate to racism: *‘diversity’, ‘social constructionism’, ‘identity’* and *‘culture*’ (BPS, 2017, p. 11) or through the more explicit guidance of the APA which notes racism, among other discriminations, is *‘highly relevant to teaching about diversity across all five learning goals of the undergraduate psychology curriculum*’ (APA, 2013, p. 13, 2019). Indeed, a wealth of resources exist to facilitate this (Fairchild, 2017; Kurtiş & Adams, 2015; Owusu‐Bempah & Howitt, 2000; Salter et al., 2018; Sonn, 2008; Wilbraham, 2016). For example, Schmidt (2019) outlines psychology teaching examples about the violence, impact, and ubiquity of Canadian colonization on human behaviour.

### University support and discrimination

Between 33% and 45% of all students did not agree they received or could access the seven different types of university support. Furthermore, BAME students reported being less able to access mitigation, extensions, and other forms of assessment support. BAME students also reported significantly more discrimination (than White students). This was largely racism, however, other discrimination types were also reported (e.g., sexism) emphasizing discrimination’s intersectional nature (Delgado & Stefancic, 2017). Sixteen percent of BAME students reported experiencing racism in the NUS research (NUS & Universities UK, 2019), whilst in the present study, 50% reported experiencing any kind of discrimination. These results eschew university’s colour‐blindness by adding to the concrete body of evidence that BAME students are disadvantaged within universities in the United Kingdom (Brown & Jones, 2013; Bunce et al., 2019; McDuff et al., 2018; NUS & Universities UK, 2019; NUS, 2012) and elsewhere (Savas, 2014; Solórzano & Yosso, 2002). Given universities typically have established support systems already in place (including assessment and mental health support), widening the accessibility of these to all students, especially BAME students, can be an immediate and significant anti‐racist gain.

Furthermore, in this study more BAME students felt unable to: share their perspectives with—and receive equal treatment from—lecturers concurring with previous research conducted almost 10 years ago (NUS, 2012). The continuing need for university staff (who tend to be White; Mahony & Weiner, 2020) to retrain in their interactions and treatment of BAME students is again underscored (NUS & Universities UK, 2019; Owusu‐Kwarteng, 2020; Solorzano, Ceja, & Yosso, 2000). Furthermore, assessing staff’s support of positive actions, as fellow university stakeholders is also important.

### Anti‐Racist recommendations in higher education

Overall, students were united in supporting the five university anti‐racist recommendations including those specifically targeted to BAME students. Encouragingly, participants did not differ in their support of the recommendations by the ‘race’ of the proposer, thus contradicting Gardner and Ryan's findings (2020)^2^. Participants also made other recommendations including the need to implement robust, zero‐tolerance, racist harassment processes (the need for which is backed up by other research; for example, Equalities & Human Rights Commission, 2019). Most of the open‐ended responses (89%) also indicated support for anti‐racism too typically encouraging more anti‐racist research and actions. These findings add to the growing body of evidence showing anti‐racist positive actions are supported by students (NUS & Universities UK, 2019; NUS, 2012) and show anti‐racism converges with university management’s interests given support for it arises from a key stakeholder (Bell, 1995; Delgado & Stefancic, 2017; Savas, 2014). Encouragingly, the concept of interest convergence, suggests that White students may have supported the recommendations because they can see that these serve their own interests (because anti‐racism means studying at a more just institution and/or because some policies may be perceived as directly benefiting white students too, e.g., anonymized marking; Salter & Adams, 2013).

Some might argue these anti‐racist recommendations do not go as far as CRT requires, given racism is institutionally embedded (Delgado & Stefancic, 2017), however, they may be important first steps in a wider programme of positive action challenging university racism. Positive action in UK universities tends to be minimal with most offering widening participation schemes only (although for exceptions to this see: Stevenson et al., 2019). These schemes typically attend to students’ geographic location and care experience but not racism (which operates ‘through’ such factors anyway: Gorard, Boliver, Siddiqui, & Banerjee, 2019; Ladson‐Billings & Tate, 1995; Warikoo & Allen, 2019). Unsurprisingly then, most participants’ positive actions likely arose from these widening participation schemes. There are three reasons why positive actions should be ‘race’ based. First because class‐based positive actions alone will not undo racism (e.g., given it is more likely to reach White working‐class students numerically; Khan & Shaheen, 2017). This is supported by the lack of access reported to some areas of university support among BAME participants. Second, because the recommendations, which were proposed by students (e.g., NUS & Universities UK, 2019), were supported by almost all student participants in this study including those recommendations that targeted BAME students only. Finally, anti‐racist positive actions may benefit White people as much as they would BAME students (e.g., by better preparing them to work in a diverse workforce; Maxwell & Garcia, 2019; Park & Liu, 2014).

The implementation of anti‐racist positive actions requires nuance to avoid the frequent charges of tokenism or reverse racism (Aguirre, 2000; López, 2003; Savas, 2014). As Peterson and Rudgers (2017, para. 16) note: ‘*We recognize that the arguments in support of affirmative action are complicated*. *They are high‐definition messages in a low‐definition world*’. Furthermore, positive action will need to be defended; as evidenced by the attacks on US anti‐racist contextual admissions (Maxwell & Garcia, 2019; Park & Liu, 2014; Tran, 2019; Warikoo & Allen, 2019). Fortunately, there is expert guidance on how to implement anti‐racist positive actions (NUS & Universities UK, 2019; Stevenson et al., 2019) and empirical evidence of their benefits (e.g., Clayton, 2012; Maxwell & Garcia, 2019; Peterson & Rudgers, 2017) that can be utilized. Psychologists, within and outside of Higher Education, can advocate here.

### Limitations

The sample was recruited via *Prolific* for a study about views on racism in exchange for financial credits. Thus although the sample was more removed from traditional samples recruited (e.g., through an author’s single institution: Bunce et al., 2019; Kanter et al., 2017; Owusu‐Kwarteng, 2020; Spanierman et al., 2008) and participation occurred anonymously where incentives were awarded regardless of response, participants who may have held more colour blind or racist views may have avoided participating in the survey, potentially leading to a more anti‐racist student view than actually exists in universities. A further limitation is the homogenization of BAME students, including international and home students, which obscures the very real inequalities within these groups (NUS & Universities UK, 2019). The sample here (*N* = 395) is a fraction of the UK student population (around 2.4 million; Universities UK, n.d) and was over representative of BAME students (48% in this sample vs. 23% in the UK university population; UK Government, 2020). Finally, whilst these survey items were constructed for purpose and based on easily comparable previous research (following the QuantCrit approach; Garcia et al., 2018; Gillborn et al., 2018), they had limitations such as not being broad enough to capture racism in its many varied forms. Thus, there is a danger that the results favour a white status quo (Gillborn et al., 2018) by overstating how informed students are about racism and underplaying how distressing BAME students’ university experiences can be. Qualitative work should be attended to those which captures these experiences (e.g., NUS & Universities UK, 2019; Owusu‐Kwarteng, 2020; Solorzano et al., 2000; Yosso, 2013). Further research should attend to the questions and topics participants suggested in the open‐ended responses (see also Table 5). These include the need to explore racism among university peer groups and staff, to assess racism witnessed (and any proxy impact this might have) and to assess racism’s intersections. These are fruitful further research directions provided by key university stakeholders who have insight into university operations: students.

### Conclusion

This study sought to understand university students’ support of key anti‐racist recommendations, students’ understanding of racism and experiences of positive actions, discrimination and university support, informed by key CRT tenets (Crenshaw, 2006; Delgado & Stefancic, 2017; Garcia et al., 2018). Findings revealed high support for the anti‐racist recommendations across students regardless of the proposer’s ‘race’. Participants also reported similar experiences of positive discrimination, relatively informed understandings of racism and access to four sources of student support. White students reported less discrimination, more access to three sources of university support and were slightly more inaccurate about racism relative to BAME students. In addition, evidence of colour‐blindness, under‐estimation of racism and ‘majoritarian stories’ that support the status quo was also found (Solórzano & Yosso, 2002; Spanierman et al., 2008). Ultimately, it is within universities’ own interests to implement these student‐stakeholder supported recommendations, especially given these steps can begin to undo the higher rates of discrimination and areas of lowered university support BAME students experience (a well‐borne finding from university research: Equalities & Human Rights Commission, 2019; NUS & Universities UK, 2019; Solorzano et al., 2000). Psychologists, particularly those within universities, can advocate for the top–down implementation of anti‐racist steps, can teach racism as a structural system in their curricula, and can help widen the accessibility of university support systems to all students, particularly BAME students. These steps will help to realize psychology’s commitment to anti‐racism (APA, 2021; BPS, 2020) and help to produce an anti‐racist, CRT‐informed, education for the benefit of all (Gillborn & Ladson‐Billings, 2010; Ladson‐Billings & Tate, 1995; Schmidt, 2019).

## Author contributions

Glen Jankowski (Conceptualization; Data curation; Formal analysis; Investigation; Methodology; Project administration; Resources; Software; Supervision; Validation; Visualization; Writing – original draft; Writing – review & editing).

## Conflict of interest

All authors declare no conflict of interest.

## Data Availability

The data that support the findings of this study are openly available in the open science framework repository at https://osf.io/rmd54/, https://doi.org/10.17605/osf.io/rmd54.

## Footnotes

^1^
BAME, as a term, and as an analysis is highly contested for homogenizing people who have diverse experiences of inequalities and for contributing to the essentialization of groups (Howarth, 2009; Hylton, 2018). Though other terms such as ‘people of colour’ are not immune to these critiques. BAME as a term is used here because it is still widely deployed by governing ‘equality’ bodies (e.g., the ONS), because it is difficult to address racism as it occurs in universities without identifying, first, its broad basis that largely advantages White students as a whole, because statistical analyses by specific ‘races’ reduce statistical power and because the study is on racism not ‘race’. The term preferred here has ‘minoritized’ rather than ‘minority’ in the acronym which highlights the active process of racialization (and disadvantage) done to those read as ‘BAME’ relative to White people following (Hylton, 2018).

^2^
There were differences between the two studies that might explain this, noticeably Gardner and Ryan (2020) invoked a Black proposer of the anti‐racist recommendations (vs. an Asian proposer), manipulated the name and photograph of the proposer (rather than a name only), and their recommendations came from a co‐worker (rather than a professor).
